# Adiposity, Fat-Free Mass Index, and Muscular Strength in Children: Independent Effects on Functional Performance in a Tertiary Pediatric Endocrinology Cohort

**DOI:** 10.3390/medicina62040730

**Published:** 2026-04-11

**Authors:** Bogdan Mihai Pascu, Ana Maria Cula, Anca Bălănescu, Paul Cristian Bălănescu, Ioan Gherghina

**Affiliations:** 1 Pediatric Department, Faculty of Medicine, University of Medicine and Pharmacy “Carol Davila”, 030167 Bucharest, Romania; 2 National Institute for Mother and Child Health “Alessandrescu-Rusescu”, 020395 Bucharest, Romania; 3 Yuno Clinic, EASO COM Obesity Center, 020459 Bucharest, Romania

**Keywords:** childhood obesity, adiposity, body composition, muscle strength, puberty, pediatric endocrinology

## Abstract

*Background and Objectives*: Childhood obesity is associated with alterations in body composition that may impair muscular strength and functional capacity. While higher body mass is often accompanied by greater absolute strength, the independent effect of adiposity on muscle strength after accounting for lean mass remains insufficiently understood. This study aimed to evaluate the associations between adiposity and muscle strength in children and adolescents, while accounting for growth and maturation, and to examine differences according to weight status. *Materials and Methods*: This retrospective cross-sectional study included 84 children and adolescents aged 5–18 years. Anthropometric measurements were used to calculate body mass index (BMI), waist-to-hip ratio, and waist-to-height ratio, with weight status classified according to CDC BMI-for-age percentiles. Body composition was assessed using bioelectrical impedance analysis (Tanita). Pubertal stage was evaluated using Tanner classification. Muscle strength was assessed using dominant handgrip strength. Associations between adiposity-related parameters and muscle strength were analyzed using correlation and multivariable linear regression models adjusted for age, sex, pubertal stage, physical activity, and body composition. *Results*: Body mass index was positively correlated with absolute handgrip strength (r = 0.561, *p* < 0.001). Body fat percentage was negatively associated with relative handgrip strength (r = −0.381, *p* < 0.001). In multivariable regression analyses, body fat percentage remained an independent negative predictor of handgrip strength (β = −0.203, *p* = 0.0046), whereas fat-free mass and fat-free mass index were positive predictors in respective models (*p* < 0.001). *Conclusions*: Increased adiposity is associated with reduced muscle strength in children and adolescents when strength is evaluated relative to body size or adjusted for lean mass. These findings support the concept of impaired muscle performance in pediatric populations with excess adiposity and highlight the importance of integrating body composition and functional assessments in clinical evaluation.

## 1. Introduction

Childhood obesity represents a major global health challenge, with increasing prevalence and well-established long-term consequences for metabolic and cardiovascular health [1,2,3]. Although excess body weight is commonly assessed using body mass index (BMI), growing evidence suggests that obesity-related health risks are more closely associated with alterations in body composition and physical function than with body weight alone.

Skeletal muscle plays a central role in metabolic regulation, insulin sensitivity, and physical performance. During childhood and adolescence, muscle mass and strength increase progressively under the influence of growth, hormonal maturation, and physical activity, with puberty representing a critical period for muscle development [4,5]. Disruptions in normal muscle development during this stage may have long-term consequences for health.

Lean tissue quantity is a major determinant of muscle strength; however, absolute fat-free mass is strongly influenced by body size and height. Fat-free mass index (FFMI), defined as fat-free mass normalized to height squared (kg/m^2^), provides a size-adjusted indicator of lean mass and may better reflect muscular development during growth. Despite its potential relevance, the role of FFMI in relation to muscle strength in pediatric populations, particularly across different stages of growth and weight status, remains incompletely characterized [6].

Children with overweight and obesity often present with increased absolute lean mass due to greater mechanical loading; however, evidence regarding muscle strength in this population is inconsistent [7,8,9,10]. While some studies report preserved or increased absolute strength, others demonstrate reduced relative strength and impaired muscle function when adjusted for body size or lean mass. Proposed mechanisms include fatty infiltration of skeletal muscle, chronic low-grade inflammation, and reduced levels of physical activity [11]. These findings suggest that the relationship between adiposity and muscle function in children remains complex, particularly when growth and pubertal development are considered.

Handgrip strength is a simple, reliable, and non-invasive marker of overall muscle strength and has been associated with cardiometabolic risk in pediatric populations [12,13,14]. However, relatively few studies have simultaneously evaluated adiposity, height-adjusted lean mass (FFMI), and both absolute and relative muscle strength across a wide pediatric age range and different pubertal stages.

The primary aim of this study was to evaluate the relationship between adiposity-related parameters and muscle strength in children and adolescents, using comprehensive anthropometric and body composition assessments while accounting for pubertal stage. The secondary aim was to examine differences in muscle strength and body composition between normal-weight children and those with overweight and obesity.

We hypothesized that increased adiposity would be associated with reduced muscle strength when expressed relative to body size or adjusted for lean mass, despite higher absolute strength in children with overweight and obesity.

## 2. Materials and Methods

### 2.1. Study Design and Participants

This study represents a retrospective cross-sectional analysis of prospectively collected clinical data from 84 consecutive children and adolescents aged 5–18 years who were evaluated during routine clinical assessments. Participants were recruited from the Department of Pediatric Endocrinology at the “Alessandrescu-Rusescu” National Institute for Mother and Child Health (INSMC), Bucharest. Recruitment was conducted over a defined period, from 15 July to 10 September 2025.

Eligibility criteria included age ≥ 5 years, which was selected to ensure reliable assessment of body composition using bioelectrical impedance analysis (Tanita), as younger children may not adequately comply with standardized measurement protocols. Additional inclusion criteria were the availability of complete anthropometric, body composition, pubertal staging, and muscle strength data.

Exclusion criteria comprised the presence of chronic diseases, acute infections, or medical conditions known to affect growth, body composition, or muscle function, including endocrine disorders other than obesity, genetic syndromes, neuromuscular diseases, and the use of medications that could influence body composition or muscle strength.

Although participants were recruited from a tertiary pediatric endocrinology center, only children without conditions affecting growth, body composition, or muscle function were included, as defined by the exclusion criteria. Thus, the study population reflects children referred for clinical evaluation rather than a disease-specific cohort.

The sample size was determined by the number of eligible participants available during the predefined recruitment period and was not based on an a priori sample size calculation.

### 2.2. Anthropometric Measurements

Height was measured to the nearest 0.1 cm using a wall-mounted stadiometer (Seca^®^, Hamburg, Germany), and body weight was measured to the nearest 0.1 kg using a calibrated digital scale (Seca^®^, Hamburg, Germany), with participants wearing light clothing and no shoes. Body mass index (BMI) was calculated as weight (kg) divided by height squared (m^2^).

BMI values were converted to age- and sex-specific percentiles using the **Centers for Disease Control and Prevention (CDC)** growth reference charts. Participants were classified as underweight (<5th percentile), normal weight (5th–84th percentile), overweight (85th–94th percentile), or obese (≥95th percentile) according to CDC criteria [15].

Waist and hip circumferences were measured using a non-elastic measuring tape (SECA, Hamburg, Germany) following standardized procedures. Waist-to-hip ratio (WHR) and waist-to-height ratio (WHtR) were calculated accordingly. Central adiposity was assessed using WHtR, with a cut-off value of ≥0.50 considered indicative of increased cardiometabolic risk in pediatric populations, independent of age and sex [16,17].

### 2.3. Pubertal Assessment

Pubertal development was assessed by a trained clinician using Tanner staging, from stage I (prepubertal) to stage V (fully mature), based on standardized clinical criteria [18,19].

### 2.4. Body Composition Analysis

Body composition was assessed using bioelectrical impedance analysis with a Tanita PRO DC430 MA device (Tanita Corporation, Tokyo, Japan). The device provided estimates of body fat percentage, fat mass, fat-free mass, muscle mass, total body water percentage, and total body water volume.

Measurements were performed according to the manufacturer’s recommendations under standardized conditions. All assessments were conducted in the morning, with participants in a fasting state, adequately hydrated, wearing light clothing, barefoot, and after bladder emptying, in order to minimize variability related to hydration status. Only children aged ≥5 years were included, as younger children may not reliably comply with standardized bioelectrical impedance measurement protocols.

Fat-free mass index (FFMI) was calculated as fat-free mass (kg) divided by height squared (m^2^). Age- and sex-specific FFMI percentile categories were derived using published pediatric reference data [7], and participants were classified as <P10, P10–P49, P50–P89, or ≥P90.

### 2.5. Muscle Strength Assessment

Muscle strength was assessed by measuring dominant handgrip strength using a calibrated handheld dynamometer (Jamar^®^, Patterson Medical, Warrenville, IL, USA). Handgrip strength was evaluated following standardized testing procedures for pediatric populations. Participants were seated with the shoulder adducted and neutrally rotated, the elbow flexed at 90°, and the forearm in a neutral position.

Three maximal voluntary contractions were performed with the dominant hand, with short rest intervals (approximately 30–60 s) between trials. Participants were instructed to squeeze the dynamometer as hard as possible during each attempt and were verbally encouraged to exert maximal effort. The highest value obtained from the three measurements was recorded and is expressed in kilograms (kg).

In addition to absolute handgrip strength, relative handgrip strength was calculated to account for differences in body size. Relative handgrip strength was defined as the ratio between absolute handgrip strength (kg) and body weight (kg), according to the following formula:

**Relative handgrip strength = handgrip strength (kg)/body weight (kg).**

This approach has been previously used to assess muscle strength relative to body mass in pediatric populations [20,21].

### 2.6. Physical Activity Assessment

Physical activity level was assessed as the average weekly hours of moderate-to-vigorous physical activity (MVPA). Information was obtained using a simple self-reported measure in older children and adolescents, or parental report for younger children, including those under 10 years of age.

Moderate-to-vigorous physical activity was defined as activities requiring at least moderate physical effort and resulting in increased heart rate and breathing, such as brisk walking, running, cycling, active play, or organized sports [22]. Weekly physical activity was recorded as the total hours of MVPA performed outside of mandatory school physical education classes.

### 2.7. Statistical Analysis

Statistical analyses were performed using **Microsoft Excel 2016** (Microsoft Corporation, Redmond, WA, USA) with the **Analysis ToolPak** add-in. Continuous variables are expressed as mean ± standard deviation (SD). Data distribution was assessed using graphical methods and the Shapiro–Wilk test for normality. Group comparisons were conducted using Student’s t-test for normally distributed variables or the Mann–Whitney U test for non-normally distributed variables, as appropriate.

Associations between adiposity-related parameters, body composition variables, and handgrip strength were evaluated using Pearson’s correlation coefficient for normally distributed data or Spearman’s rank correlation coefficient when normality assumptions were not met.

Multivariable linear regression analyses were performed to assess independent determinants of muscle strength. Absolute dominant handgrip strength (kg) and relative handgrip strength (handgrip strength/body weight) were used as dependent variables in separate models. Independent variables were selected a priori based on biological plausibility and previous literature and included age, sex, pubertal stage (Tanner stage), weekly physical activity, and measures of adiposity and body composition. No automated variable selection procedures were applied.

Three regression models were constructed:

**Model A:** Absolute handgrip strength (kg) as the dependent variable, including body fat percentage and fat-free mass as key predictors.

**Model B:** Relative handgrip strength (handgrip strength/body weight) as the dependent variable, including body fat percentage and covariates.

**Model C:** Absolute handgrip strength (kg) as the dependent variable, including **fat-free mass index (FFMI)** instead of absolute fat-free mass, to account for body size and height.

All models were adjusted for age, sex, pubertal stage, and physical activity. A two-sided ***p*-value < 0.05** was considered statistically significant.

Potential multicollinearity between predictors was assessed using correlation matrices and variance inflation factors (VIFs). Highly correlated variables were not included simultaneously in the same model. In particular, fat-free mass and FFMI were analyzed in separate models to avoid redundancy.

All variables had complete data, and no imputation was required.

Model assumptions were assessed by visual inspection of residual plots to evaluate linearity and homoscedasticity, and by examining the distribution of residuals to confirm normality.

### 2.8. Ethical Approval

The study was conducted in accordance with the Declaration of Helsinki and was approved by the Ethics Committee of the National Institute for Mother and Child Health “Alessandrescu-Rusescu” (approval code no. 52/3 January 2023). Given the retrospective nature of the study and the use of anonymized data extracted from electronic medical records, the requirement for informed consent was waived by the Ethics Committee.

### 2.9. Data Availability

The data supporting the findings of this study are available from the corresponding author upon reasonable request. The dataset has not been deposited in a public repository due to ethical and privacy considerations involving pediatric participants.

### 2.10. Use of Generative Artificial Intelligence

Generative artificial intelligence was not used for study design, data collection, data analysis, or data interpretation. AI-based tools were used solely for language editing and formatting of the manuscript.

## 3. Results

### 3.1. Characteristics of the Study Population

A total of 84 children and adolescents aged between 5 and 18 years were included in the study, comprising 27 females (32.1%) and 57 males (67.9%). Baseline characteristics were analyzed according to pubertal status, weight status, and sex.

Baseline characteristics according to pubertal status (prepubertal vs. pubertal) are presented in Table 1A, characteristics according to weight status are shown in Table 1B, and sex-specific characteristics are summarized in Table 1C.

### 3.2. Weight Status and Body Composition

Based on CDC BMI-for-age percentile classification, 5 participants (6.0%) were classified as underweight, 42 (50.0%) as normal weight, and 37 (44.0%) as overweight or obese. Given the small number of underweight participants, comparative analyses focused on the normal-weight and overweight/obesity groups.

Children with overweight/obesity presented higher adiposity-related parameters compared with normal-weight participants, including body fat percentage (30.74 ± 8.96% vs. 14.92 ± 4.60%, *p* < 0.001) and fat mass (22.19 ± 16.01 vs. 5.14 ± 2.73 kg, *p* < 0.001). Central adiposity indices were also higher in the overweight/obesity group, including waist circumference (84.93 ± 17.27 vs. 58.10 ± 9.93 cm, *p* < 0.001) and waist-to-height ratio (0.53 ± 0.08 vs. 0.42 ± 0.04, *p* < 0.001). Fat-free mass (46.88 ± 14.25 vs. 29.88 ± 13.20 kg, *p* < 0.001) and FFMI (18.01 ± 2.62 vs. 14.33 ± 2.18 kg/m^2^, *p* < 0.001) were higher in the overweight/obesity group. Differences in body composition and adiposity-related parameters according to weight status are summarized in Table 2.

### 3.3. Handgrip Strength: Absolute Versus Relative Measures

Absolute dominant handgrip strength differed between weight status groups. Children with overweight and obesity presented higher mean absolute handgrip strength compared with normal-weight participants (22.01 ± 10.50 kg vs. 13.36 ± 10.02 kg; *p* < 0.001).

When handgrip strength was expressed relative to body weight, relative handgrip strength values were lower in children with overweight and obesity compared with normal-weight participants (0.32 ± 0.12 vs. 0.34 ± 0.15), with no statistically significant difference between groups (*p* = 0.384).

**Figure 1:**
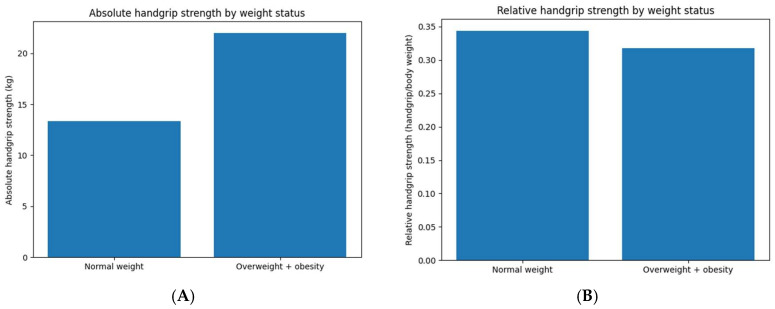
Comparison of absolute (**A**) and relative (**B**) dominant handgrip strength between normal-weight children and overweight/obesity children.

Differences between absolute and relative handgrip strength measures according to weight status are presented in Table 2 and illustrated in Figure 1.

### 3.4. Correlation Analyses

Correlation analyses revealed strong positive associations between dominant handgrip strength and growth- and maturation-related variables, including age (r = 0.697, *p* < 0.001), height (r = 0.828, *p* < 0.001), and pubertal stage (r = 0.731, *p* < 0.001). Fat-free mass showed the strongest positive correlation with handgrip strength (r = 0.885, *p* < 0.001).

Positive correlations were also observed between handgrip strength and BMI (r = 0.561, *p* < 0.001) as well as fat mass (r = 0.347, *p* = 0.001).

**Figure 2:**
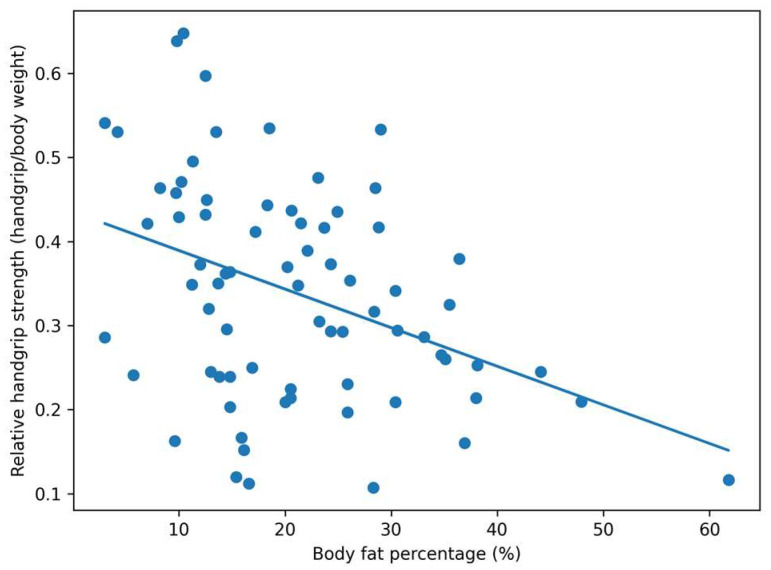
Association between body fat percentage and relative handgrip strength (handgrip strength/body weight ratio) in the study population.

In contrast, body fat percentage was negatively correlated with relative handgrip strength (handgrip strength/body weight ratio) (r = −0.381, *p* < 0.001). This relationship is illustrated in Figure 2.

**Figure 3:**
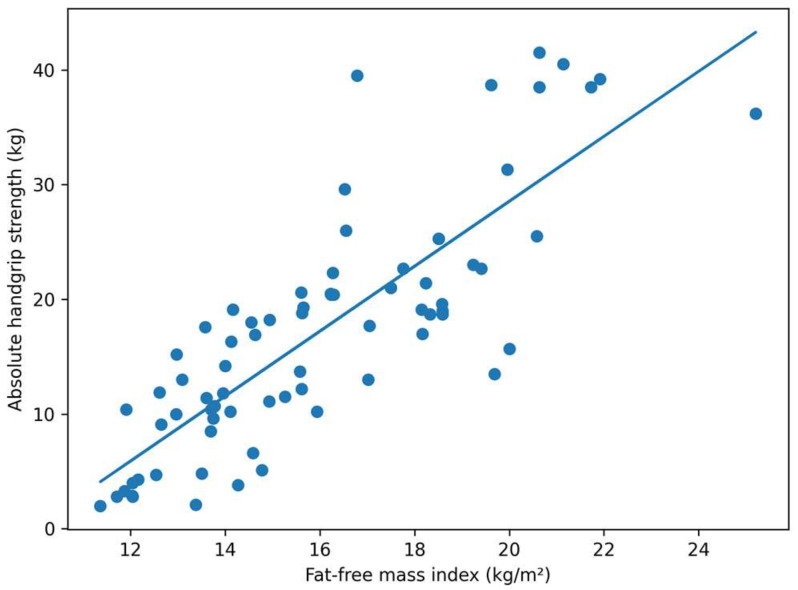
Association between fat-free mass index (FFMI) and absolute dominant handgrip strength in the study population.

Fat-free mass index also showed a positive association with absolute handgrip strength. This relationship is illustrated in Figure 3.

### 3.5. Multivariable Regression Analyses

Multivariable linear regression models were constructed to examine associations between adiposity-related parameters, body composition variables, and handgrip strength outcomes.

In Table 3A, with absolute handgrip strength as the dependent variable and adjusted for age, sex, Tanner stage, physical activity, and fat-free mass, body fat percentage was negatively associated with handgrip strength (β = −0.203 kg per 1% increase in body fat, *p* = 0.0046). Fat-free mass was positively associated with handgrip strength (β = 0.707 kg per 1 kg increase, *p* < 0.001). The model explained 83.4% of the variance in absolute handgrip strength (R^2^ = 0.834).

In Table 3B, using relative handgrip strength (handgrip strength/body weight) as the dependent variable and adjusted for age, sex, Tanner stage, and physical activity, body fat percentage was negatively associated with relative handgrip strength (β = −0.00412 per 1% increase in body fat, *p* = 0.00064).

In Table 3C, fat-free mass was replaced by fat-free mass index (FFMI). FFMI was positively associated with absolute handgrip strength (β = 2.21 kg per 1 kg/m^2^ increase, *p* < 0.001), while Tanner stage was also associated with handgrip strength. Body fat percentage was not significantly associated with absolute handgrip strength in this model.

Results of the multivariable regression analyses are summarized in Table 3.

### 3.6. Fat-Free Mass Index and Muscle Strength

Fat-free mass index (FFMI) differed according to weight status. Children with overweight and obesity presented higher mean FFMI values compared with normal-weight participants (18.01 ± 2.62 vs. 14.33 ± 2.18 kg/m^2^, respectively) (Table 4).

FFMI was positively associated with absolute dominant handgrip strength. This association is illustrated in Figure 3, showing a positive association between FFMI and absolute handgrip strength.

Analysis of FFMI percentile categories showed differences in the distribution of FFMI according to weight status (Table 4). A higher proportion of children with overweight and obesity were classified in the ≥P90 FFMI category, whereas most normal-weight participants were distributed within the P10–P49 percentile range.

In multivariable regression analyses in which fat-free mass was replaced by FFMI, FFMI was positively associated with absolute handgrip strength after adjustment for age, sex, pubertal stage, physical activity, and body fat percentage (Table 3).

## 4. Discussion

The present study examined the relationship between adiposity, body composition, and muscle strength in a pediatric population spanning a wide age range and different stages of pubertal development. The main finding was that increased adiposity was associated with reduced muscle strength when strength was expressed relative to body mass or evaluated after adjustment for lean tissue, despite higher absolute handgrip strength observed in children with overweight and obesity. These findings suggest a dissociation between muscle quantity and muscle quality in pediatric populations with excess adiposity, a concept increasingly recognized in obesity research [23].

In agreement with previous reports, children with overweight and obesity in our cohort exhibited higher absolute handgrip strength than their normal-weight peers, a finding largely attributable to greater body size and increased fat-free mass [9,10]. Increased mechanical loading associated with excess body weight may contribute to higher absolute muscle mass during growth. However, this apparent advantage did not translate into improved functional performance when strength was normalized to body weight or when adiposity-related parameters were included in multivariable models.

An important contribution of the present study is the inclusion of fat-free mass index (FFMI) as a height-adjusted marker of lean tissue. FFMI showed a strong positive association with absolute handgrip strength and remained a significant predictor in multivariable analyses. These findings suggest that FFMI may represent a useful marker of lean mass during growth. At the same time, adiposity-related parameters remained negatively associated with relative strength outcomes, indicating that excess adiposity may be accompanied by reduced muscle performance relative to body size.

FFMI may provide clinically relevant information beyond BMI and absolute fat-free mass by helping to distinguish between children with elevated BMI due to increased lean mass and those with elevated BMI associated predominantly with excess adiposity. This distinction may be particularly relevant in pediatric populations, in whom growth-related changes in body composition can complicate the interpretation of BMI alone [6].

The inverse association between body fat percentage and relative muscle strength observed in this study is consistent with previous literature describing impaired muscle function in children with obesity [10,15]. Several mechanisms may contribute to this relationship, as previously described in studies addressing muscle quality in obesity [23]. Fat infiltration into skeletal muscle may alter muscle architecture and reduce contractile efficiency, while obesity-related low-grade inflammation may impair muscle metabolism and regenerative capacity [11]. In addition, lower habitual physical activity levels, frequently reported in pediatric populations with excess adiposity, may further contribute to diminished functional performance.

Pubertal development emerged as a major determinant of muscle strength, as reflected by the strong associations between Tanner stage and handgrip strength. Importantly, the negative associations between adiposity and muscle strength persisted after adjustment for pubertal stage, suggesting that these effects are not explained solely by differences in biological maturation.

Handgrip strength is increasingly recognized as a practical and reliable marker of overall muscle function and has been associated with cardiometabolic risk in pediatric populations [12,13]. The present findings further support its use as a functional outcome measure in studies of childhood overweight and obesity. Incorporating muscle strength assessment alongside traditional anthropometric indices and detailed body composition measures, including FFMI, may provide a more comprehensive evaluation of health status in children with excess adiposity.

Recent studies using bioelectrical impedance vector analysis (BIVA) have shown that parameters such as vector length and phase angle may provide additional information on muscle quality and cellular integrity [24], further supporting the concept that muscle quantity and muscle function may be dissociated in obesity. Although BIVA was not applied in the present study, these data are in line with the broader interpretation of bioelectrical impedance-derived markers beyond BMI alone.

Several limitations should be acknowledged. First, the cross-sectional design precludes causal inference regarding the relationship between adiposity and muscle strength. Second, the study included a relatively modest sample size recruited from a tertiary pediatric endocrinology center, which may introduce referral bias and limit the generalizability of the findings to the broader pediatric population. The unequal sex distribution and the wide age range of participants may also have influenced strength outcomes, despite statistical adjustment for pubertal stage. Third, body composition was assessed using bioelectrical impedance analysis rather than imaging techniques such as dual-energy X-ray absorptiometry or magnetic resonance imaging and therefore did not allow direct evaluation of muscle quality or intramuscular adipose tissue. In addition, physical activity was assessed through self- or parent-reported data, which may be subject to recall bias and social desirability bias. This limitation may have reduced the ability to detect true associations between physical activity and muscle strength and may have contributed to residual confounding in the regression analyses. Finally, muscle strength assessment relied solely on handgrip dynamometry and may not fully reflect global muscular performance.

Overall, these findings emphasize that childhood overweight and obesity should not be evaluated solely on the basis of body weight or BMI. Functional measures such as muscle strength, together with height-adjusted indices of lean mass, may provide additional insight into the health consequences of excess adiposity during growth.

## 5. Conclusions

In this retrospective cross-sectional study, increased adiposity was associated with reduced muscle strength in children and adolescents when strength was evaluated relative to body mass or adjusted for lean tissue. Although children with overweight and obesity exhibited higher absolute handgrip strength, this finding was largely explained by greater fat-free mass and overall body size. Measures of adiposity, particularly body fat percentage, remained negatively associated with functional strength outcomes after adjustment for age, sex, pubertal stage, physical activity, and body composition.

These results underscore the dissociation between muscle quantity and muscle quality in pediatric obesity and highlight the clinical relevance of functional muscle assessment. Integrating body composition analysis with simple strength measurements, such as handgrip strength, may improve the evaluation of obesity-related functional impairment and help guide more targeted interventions during growth.

## Figures and Tables

**Table 1:** (**A**). Baseline characteristics according to pubertal status. (**B**). Baseline characteristics according to weight status. (**C**). Baseline characteristics according to sex.

(**A**)			
**Variable**		**Prepubertal (Tanner I) (*n* = 27)**	**Pubertal (Tanner II–V) (*n* = 57)**
Age (years)		7.78 ± 2.31	13.46 ± 2.32
Height (cm)		124.87 ± 16.11	159.91 ± 12.61
Weight (kg)		28.67 ± 14.67	59.66 ± 24.57
Body mass index (kg/m^2^)		17.24 ± 4.30	22.64 ± 6.50
Waist circumference (cm)		58.22 ± 14.22	75.03 ± 18.95
Hip circumference (cm)		66.69 ± 14.34	89.61 ± 15.89
Waist-to-hip ratio		0.87 ± 0.08	0.83 ± 0.11
Waist-to-height ratio		0.46 ± 0.07	0.47 ± 0.09
Physical activity (h/week)		1.22 ± 1.77	2.84 ± 2.79
Body fat (%)		20.00 ± 9.44	21.93 ± 11.73
Fat mass (kg)		6.76 ± 6.66	15.12 ± 15.51
Fat-free mass (kg)		21.93 ± 8.70	44.61 ± 13.04
Total body water (%)		58.53 ± 6.92	57.25 ± 8.70
Total body water (kg)		16.04 ± 6.38	32.71 ± 9.57
Muscle mass (%)		20.71 ± 8.30	42.33 ± 12.42
Fat-free mass index (FFMI, kg/m^2^)		13.45 ± 1.72	17.05 ± 2.79
Dominant handgrip strength (kg)		6.98 ± 4.43	21.95 ± 9.64
Relative handgrip strength (handgrip/body weight)		0.24 ± 0.11	0.38 ± 0.12
(**B**)			
**Variable**	**Underweight (*n* = 5)**	**Normal Weight (*n* = 42)**	**Overweight + Obesity (*n* = 37)**
Age (years)	12.60 ± 3.05	10.79 ± 3.84	12.46 ± 3.01
Height (cm)	145.22 ± 20.10	139.91 ± 22.85	159.02 ± 14.81
Weight (kg)	30.50 ± 9.95	35.01 ± 15.05	68.97 ± 25.10
Body mass index (kg/m^2^)	14.01 ± 1.51	16.85 ± 2.44	26.44 ± 5.45
Waist circumference (cm)	53.20 ± 6.30	58.10 ± 9.93	84.93 ± 17.27
Hip circumference (cm)	66.40 ± 9.91	71.06 ± 13.26	97.07 ± 13.81
Waist-to-hip ratio	0.81 ± 0.07	0.83 ± 0.11	0.87 ± 0.09
Waist-to-height ratio	0.37 ± 0.03	0.42 ± 0.04	0.53 ± 0.08
Physical activity (h/week)	2.60 ± 3.97	2.61 ± 3.76	1.96 ± 2.44
Body fat (%)	5.24 ± 2.83	14.92 ± 4.60	30.74 ± 8.96
Fat mass (kg)	1.44 ± 0.61	5.14 ± 2.73	22.19 ± 16.01
Fat-free mass (kg)	29.06 ± 10.04	29.88 ± 13.20	46.88 ± 14.25
Total body water (%)	69.40 ± 2.07	62.41 ± 3.56	50.69 ± 6.55
Total body water (kg)	21.28 ± 7.35	21.94 ± 9.80	34.31 ± 10.43
Muscle mass (%)	27.50 ± 9.53	28.28 ± 12.58	44.50 ± 13.58
Fat-free mass index (FFMI, kg/m^2^)	13.29 ± 1.63	14.33 ± 2.18	18.01 ± 2.62
Dominant handgrip strength (kg)	12.78 ± 6.49	13.36 ± 10.02	22.01 ± 10.50
Relative handgrip strength (handgrip/body weight)	0.40 ± 0.16	0.34 ± 0.15	0.32 ± 0.12
(**C**)			
**Variable**		**Girls (*n* = 27)**	**Boys (*n* = 57)**
Age (years)		11.37 ± 3.47	11.75 ± 3.57
Height (cm)		145.95 ± 17.47	149.92 ± 23.12
Weight (kg)		46.31 ± 19.15	51.31 ± 28.98
Body mass index (kg/m^2^)		20.80 ± 5.73	20.95 ± 6.72
Waist circumference (cm)		66.24 ± 13.97	71.23 ± 21.13
Hip circumference (cm)		82.09 ± 16.10	82.31 ± 19.98
Waist-to-hip ratio		0.81 ± 0.09	0.86 ± 0.10
Waist-to-height ratio		0.45 ± 0.08	0.47 ± 0.09
Physical activity (h/week)		1.94 ± 3.71	2.50 ± 2.99
Body fat (%)		25.47 ± 10.62	19.34 ± 10.75
Fat mass (kg)		13.35 ± 9.69	11.99 ± 15.48
Fat-free mass (kg)		32.99 ± 10.76	39.37 ± 17.50
Total body water (%)		54.53 ± 7.75	59.15 ± 7.98
Total body water (kg)		24.14 ± 7.89	28.87 ± 12.85
Muscle mass (%)		31.30 ± 10.24	37.31 ± 16.70
Fat-free mass index (FFMI, kg/m^2^)		14.96 ± 2.18	16.33 ± 3.27
Dominant handgrip strength (kg)		13.59 ± 7.53	18.82 ± 11.84
Relative handgrip strength (handgrip/body weight)		0.29 ± 0.12	0.36 ± 0.13

Data are presented as mean ± standard deviation.

**Table 2:** Body composition and adiposity parameters according to weight status.

Variable	Normal Weight (*n* = 42)	Overweight + Obesity (*n* = 37)	*p*-Value
Body mass index (kg/m^2^)	16.85 ± 2.44	26.44 ± 5.45	<0.001
Waist circumference (cm)	58.10 ± 9.93	84.93 ± 17.27	<0.001
Hip circumference (cm)	71.06 ± 13.26	97.07 ± 13.81	<0.001
Waist-to-hip ratio	0.83 ± 0.11	0.87 ± 0.09	0.008
Waist-to-height ratio	0.42 ± 0.04	0.53 ± 0.08	<0.001
Body fat (%)	14.92 ± 4.60	30.74 ± 8.96	<0.001
Fat mass (kg)	5.14 ± 2.73	22.19 ± 16.01	<0.001
Fat-free mass (kg)	29.88 ± 13.20	46.88 ± 14.25	<0.001
Fat-free mass index (FFMI, kg/m^2^)	14.33 ± 2.18	18.01 ± 2.62	<0.001
Absolute handgrip strength (kg)	13.36 ± 10.02	22.01 ± 10.50	<0.001
Relative handgrip strength (handgrip/body weight)	0.34 ± 0.15	0.32 ± 0.12	0.384

Data are presented as mean ± standard deviation. Comparisons were performed between normal-weight and overweight/obesity groups.

**Table 3:** Multivariable linear regression models for handgrip strength. (**A**) Absolute handgrip strength (kg) as the dependent variable. R^2^ = 0.834. (**B**) Relative handgrip strength as the dependent variable (handgrip/body weight). (**C**) Absolute handgrip strength (kg) as the dependent variable with FFMI. R^2^ = 0.779, Adj. R^2^ = 0.759.

(**A**)			
**Predictor**	**β**	**95% CI**	***p*** **-Value**
**Body fat (%)**	**−0.203**	**−0.341 to −0.064**	**0.0046**
**Fat-free mass (kg)**	**0.707**	**0.537 to 0.876**	**<0.001**
Age (years)	0.182	−0.041 to 0.405	0.11
**Male sex**	**2.41**	**0.92 to 3.90**	**0.002**
**Tanner stage**	**1.36**	**0.71 to 2.01**	**<0.001**
Physical activity (h/week)	0.21	−0.05 to 0.47	0.11
(**B**)			
**Predictor**	**β**	**95% CI**	***p*** **-Value**
**Body fat (%)**	**−0.00412**	**−0.00643 to −0.00182**	**0.00064**
**Male sex**	**0.031**	**0.011 to 0.051**	**0.003**
**Tanner stage**	**0.018**	**0.006 to 0.030**	**0.004**
Physical activity (h/week)	0.003	−0.001 to 0.007	0.09
(**C**)			
**Predictor**	**β**	**95% CI**	***p*** **-Value**
Body fat (%)	−0.088	−0.253 to 0.077	0.291
**FFMI (kg/m^2^)**	**2.214**	**1.367 to 3.060**	**<0.001**
Age (years)	−0.343	−1.081 to 0.395	0.357
Male sex	2.874	−0.510 to 6.259	0.095
**Tanner stage**	**2.698**	**1.243 to 4.153**	**<0.001**
Physical activity (h/week)	0.396	−0.058 to 0.850	0.086

Bold values indicate statistically significant associations (*p* < 0.05).

**Table 4:** Fat-free mass index values and percentile categories according to weight status.

Variable	Normal Weight (*n* = 42)	Overweight + Obesity (*n* = 37)
**FFMI (kg/m^2^)**	**14.33 ± 2.18**	**18.01 ± 2.62**
FFMI < P10, n (%)	0 (0.0)	0 (0.0)
FFMI P10–P49, n (%)	39 (92.9)	2 (5.4)
FFMI P50–P89, n (%)	3 (7.1)	19 (51.4)
FFMI ≥ P90, n (%)	0 (0.0)	16 (43.2)

Data are presented as mean ± standard deviation; Bold values indicate statistically significant associations (*p* < 0.05).

## Data Availability

The original contributions presented in this study are included in the article. Further inquiries can be directed to the corresponding author.
